# Genome-wide association study and evolutionary analysis of the *CrRLK1L* family reveal *BnCrRLK1L1_5* as a positive regulator of *Sclerotinia sclerotiorum* resistance in *Brassica napus*

**DOI:** 10.1186/s12870-026-08872-5

**Published:** 2026-05-08

**Authors:** Tongyu Fu, Rong Zuo, Jie Liu, Zetao Bai, Cong Zhou, Junyan Huang, Li Ren, Yueying Liu, Fan Liu, Chaobo Tong, Li Xu, Lijiang Liu, Shengyi Liu

**Affiliations:** The Key Laboratory of Biology and Genetic Improvement of Oil Crops, Ministry of Agriculture and Rural Affairs of the PRC, Oil Crops Research Institute Chinese Academy of Agricultural Sciences, Wuhan, 430062 China

**Keywords:** CrRLK1L, *FER*, *Sclerotinia sclerotiorum*, GWAS, *Brassica napus*

## Abstract

**Background:**

Sclerotinia stem rot, caused by the necrotrophic pathogen *Sclerotinia sclerotiorum* (*S. sclerotiorum*), poses a significant threat to rapeseed (*Brassica napus*), resulting in substantial yield losses and economic damage worldwide. While receptor-like kinases, particularly the *Catharanthus roseus* RLK1-like (*CrRLK1L*) family, are known to play vital roles in plant immunity and development, their specific functions in *B. napus* defense against *S. sclerotiorum* and their evolutionary dynamics remain largely unexplored.

**Results:**

Through a GWAS, we identified *BnCrRLK1L1_5*, a *CrRLK1L* family member, as a candidate gene conferring resistance to *S. sclerotiorum*. To understand its evolutionary context, genome-wide and phylogenetic analyses of the *CrRLK1L* family across three *Brassica* species were performed, revealing significant diversification driven by whole-genome duplications and gene loss. Nevertheless, key functional domains, such as the malectin-like and kinase domains, remained highly conserved. Further analysis of *cis*-elements and the identification of six *BnCrRLK1L1* paralogs in *B. napus* suggested potential functional redundancy and broad roles in mediating stress responses. Transcriptomic analysis revealed that the expression of *BnCrRLK1L1_5* was strongly induced in resistant genotypes upon inoculation. Functional characterization using *fer-4*, a mutant of the *Arabidopsis* ortholog *FERONIA* (*FER*), showed enhanced susceptibility to *S. sclerotiorum*. Furthermore, complementation with *BnCrRLK1L1_5* in the *fer-4* background restored resistance to levels exceeding the wild-type, indicating functional conservation between *BnCrRLK1L1_5* and *FER*. Co-expression of BnCrRLK1L1_5 with BAX in *N. benthamiana* leaves significantly reduced necrosis and ion leakage, suggesting that *BnCrRLK1L1_5* functions as a suppressor of cell death. Moreover, protein-protein interaction network analysis predicted interactions between BnCrRLK1L1_5 and key partners, including BnCrRLK1L1_4, PTM9, RALF23, and Ferritin 4. These findings suggest that *BnCrRLK1L1_5* modulates resistance through regulating cell wall integrity, oxidative stress, and immune signaling.

**Conclusions:**

Together, this study elucidates the dynamic evolution of the CrRLK1L family and establishes *BnCrRLK1L1_5* as a key regulator of resistance against *S. sclerotiorum.* The potential mechanisms involving immune signaling and cell death regulation provide valuable insights for improving disease resistance in rapeseed and other crops.

**Supplementary Information:**

The online version contains supplementary material available at 10.1186/s12870-026-08872-5.

## Background

The *Catharanthus roseus* receptor-like kinase 1-like (CrRLK1L) family is a prominent subfamily of the RLK superfamily that regulates key developmental processes and mediates responses to diverse stress conditions [1]. The CrRLK1L family was first identified in *Catharanthus roseus* and is characterized by an N-terminal extracellular domain, a single transmembrane helix, and a C-terminal kinase tail [2]. This structure allows CrRLK1L proteins to function as signal transducers at the plasma membrane, perceiving external signals through interactions between the extracellular malectin-like domain and RALF peptides [3].

In *Arabidopsis thaliana*, the 17 identified CrRLK1L members are grouped into 10 clades, each with distinct functions in regulating diverse biological processes [4]. Among them, *CrRLK1L1*, also known as *FERONIA* (*FER*), exhibits ubiquitous expression in vegetative and reproductive organs and plays a multifaceted role from growth to immunity [5–7]. It has been implicated in diverse developmental processes, including root hair elongation [8], flowering time [9], pollen tube growth [10], and seed development [11]. *FER* also participates in abiotic stress responses such as cold, heat, drought, and salinity [7]. It regulates cell growth and stress responses by modulating the balance of ROS and hormone homeostasis, particularly auxin, jasmonic acid, brassinosteroids, ethylene, and salicylic acid [12].

Most of all, *FER* plays a dual role in plant immunity. It positively regulates basal immune responses by forming receptor complexes with PRRs [13]. For example, overexpression of the extracellular malectin-like domain of *TaFER* enhanced resistance to *Fusarium graminearum* [14]. Moreover, FER attenuates bacterial virulence efficiency. It mediates resistance to *Pseudomonas syringae* by suppressing JA and coronatine through the phosphorylation and destabilization of MYC2 [15]. However, this pathway can be exploited by pathogens. RALF23 binds FER to stabilize MYC2 or to block immune complexes, thereby suppressing plant defense and promoting infection [13, 15].

*Brassica napus* L. is a major global oilseed crop used in food, feed, and biofuel production [16]. As an allotetraploid species (AACC) that emerged from the natural crossing between *Brassica rapa* (AA) and *Brassica oleracea* (CC), *B. napus* exhibits a complex genome [17]. However, it faces numerous biotic stresses, among which Sclerotinia stem rot caused by the necrotrophic pathogen *S. sclerotiorum* is a major threat [18]. This pathogen infects multiple tissues and persists in soil as sclerotia for years, resulting in yield and quality losses worldwide.

To date, no *B. napus* accession possesses complete immunity to *S. sclerotiorum*. Despite the identification of various resistance-associated QTLs, the isolation of a single gene conferring complete resistance remains lacking [19–23]. It implicates the complexity of resistance mechanisms and poses significant challenges for breeding efforts. Recent advances in genomics, especially GWAS, have been utilized to explore candidate resistant QTLs; only a few genes have been functionally validated. For example, *BnaA07.MKK9* enhanced Sclerotinia stem rot resistance by promoting H_2_O_2_ accumulation and hypersensitive response [24], while *BnaA07.WRKY40* regulated downstream defense-related genes in response to *S. sclerotiorum* infection [25]. Considering the devastating impact of Sclerotinia stem rot, the limited number of resistance genes identified so far has significantly hindered progress in breeding for resistance.

In this study, we integrated GWAS with functional genomics to identify *BnCrRLK1L1_5* as a critical enhancer of resistance to *S. sclerotiorum*. Using a natural population of 180 *B. napus* accessions, *BnCrRLK1L1_5* was identified as a candidate gene associated with stem rot resistance. Then, the study investigated the functional role of *BnCrRLK1L1_5* by complementing the corresponding *Arabidopsis* mutant *fer-4*. Furthermore, we performed a comprehensive genome-wide analysis of the CrRLK1L family in rapeseed, including phylogenetic and synteny analyses, gene structure and motif characterization, *cis*-element prediction, expression profiling, and protein-protein interaction networks. Together, this study investigated the functional and evolutionary roles of *CrRLK1L* genes, offering novel insights into the molecular basis of immunity against *S. sclerotiorum* in *B. napus*. It will also uncover new resistance genes, broadening the genetic resources available for combating *S. sclerotiorum*.

## Materials and methods

### Plant materials and fungal pathogens

The natural populations employed for GWAS consisted of 180 *B. napus* accessions, including spring, winter, and semi-winter types of rapeseed oil, all cultivated under natural conditions in our laboratory’s Yangluo experimental field in Wuhan, Hubei Province (Table S1). *A. thaliana* wild-type and the *fer-4* mutant were maintained in our laboratory and grown in a controlled greenhouse at 22 °C, under a 16 h light/8 h dark photoperiod. The *S. sclerotiorum* isolate 1980 was propagated on PDA at 24 °C, and its mycelium was subsequently used for further inoculation.

### Stem and leaf inoculation with *S. sclerotiorum*

Stem inoculation was conducted on 180 natural accessions of *B. napus* grown in the field during the peak flowering stage of the majority of the population. Each accession was planted in three replicated plots, with ten plants per replicate. Mycelial plugs of *S. sclerotiorum* were prepared by punching 8 mm discs from the actively growing edge of fungal cultures. A hole of the same diameter was pre-drilled into the stem of each plant, and the 8 mm mycelial plug was placed at the wound site. Then the inoculated stem was wrapped with plastic film to maintain moisture and promote infection. Each plot received three sets of inoculated plants, and lesion size was measured at 17 days post-inoculation [26]. The average lesion size from the three replicates was used as the phenotypic value for GWAS analysis.

Leaf inoculation was performed on *B. napus* cultivars ZS11 and ZY821 grown in the field during the wintering period. An 8 mm mycelial agar disc was placed at the leaf center. The size of each lesion was determined by measuring two perpendicular diameters and calculated as the mean of these values at 24 and 42 h after inoculation. Subsequently, the final “average lesion size” for each specific accession was calculated as the statistical mean of the lesion sizes across all replicates. Similarly, Arabidopsis wild-type and *fer-4* mutant were inoculated using a 2 mm mycelial plug on each leaf collected from eight-week-old plants. Lesion size was measured and calculated using the same method at 42 h after inoculation. Each accession was inoculated in three replicated plots, with ten leaves per replicate. The average lesion size from the three replicates served as the phenotypic value. Statistical significance was assessed using Student’s *t*-test.

### GWAS in a natural population

We conducted GWAS on a collection of 180 *B. napus* accessions, utilizing whole-genome resequencing data previously generated in our laboratory [27]. The Illumina HiSeq XTen platform was used for sequencing, followed by the alignment against the *B. napus* Darmor-bzh reference assembly (version 5.0). SNPs with a MAF < 0.05 were filtered out, resulting in 2,797,642 high-quality SNPs for GWAS analysis. The GWAS was implemented using both MLM and GLM. A significance cutoff of *p* < 1.8 × 10⁻⁸ was adopted in this study, corresponding to a Bonferroni correction (0.05/total number of SNPs) [28]. LDBlockShow was employed to generate heatmaps representing linkage disequilibrium and haplotype blocks in specific regions [29]. Box-and-whisker plots of phenotypic differences among haplotypes were generated based on significant SNPs via a two-tailed Student’s *t*-test.

### Genetic complementation assays in *Arabidopsis*

The complementation vector was constructed for transformation into *Arabidopsis* to validate the candidate gene. The genomic sequence of *BnCrRLK1L1_5* was amplified from *B. napus* cv. ZY821 via PCR (primers in Table S2) and inserted into the pBI121 under the control of its native promoter. The constructed vector was then electroporated into *Agrobacterium tumefaciens* strain GV3101. *Arabidopsis* mutant (*fer-4*) was transformed using the floral dip method. After selection on kanamycin-containing medium and confirmation by PCR, homozygous T₂ lines were used for leaf inoculation.

### Subcellular localization, cell death, and ion leakage measurement in *Nicotiana benthamiana*

The pBI121-mCherry vector carrying *BnCrRLK1L1_5* (primers in Table S2) was transformed into *A. tumefaciens* GV3101. For transient expression assays, bacteria were resuspended to a final OD_600_ of 0.5 in a solution of 10 mM MgCl₂, 10 mM MES (pH 5.6), and 100 µM acetosyringone. The bacterial suspensions were infiltrated into the 4- to 6-week-old *N. benthamiana* leaves using a needleless syringe [30]. Subcellular localization of *BnCrRLK1L1_5* was visualized by detecting mCherry fluorescence using confocal laser scanning microscopy after 48 h.

To assess cell death induction or inhibition, the *BnCrRLK1L1_5* construct, control vectors, and BAX were expressed in *N. benthamiana* leaves as described above. Cell death symptoms were monitored visually and documented 3 days post-infiltration. Leaf discs with a diameter of 1 cm were prepared from both inoculated and uninoculated *N*. *benthamiana* leaves using a sterile cork borer. The leaf discs were immediately immersed in 20 mL of sterile distilled water and incubated for 30 min. Initial electrical conductivity (EC_1_) of the samples and the blank control (EC_0_) was measured using an OHAUS STARTER 300 C conductivity meter. Subsequently, the samples were boiled for 30 min to achieve complete electrolyte release, then cooled to room temperature (25 °C). The total electrical conductivity (EC_2_) was then measured. The leakage percentage was calculated using the equation: (EC_1_−EC_0_) / (EC_2_−EC_0_) × 100% [31].

### Identification of CrRLK1L family members in three *Brassica* species

Sequences of the 17* A. thaliana* (Columbia_TAIR10.1) CrRLK1L members were acquired from TAIR. The genome and protein data of *B. napus* (Darmor-bzh_v5), *B. oleracea* (JZS_v2), and *B. rapa* (Chiifu_v4.1) were retrieved from BnaOmics (https://bnaomics.ocri-genomics.net/) [32].

The *A. thaliana* CrRLK1L amino acid sequences were employed as query probes to screen for homologous genes in three *Brassica* species. All CrRLK1L protein kinases are defined by a conserved architecture comprising a malectin-like domain and a kinase domain. Domain structures of the 17 Arabidopsis CrRLK1L proteins were analyzed using Pfam, which confirmed that all of them contain the malectin-like domain (Pfam ID: PF12819). Notably, ATCrRLK1L14 (AT5G39020) included the Pkinase domain (Pfam ID: PF00069), whereas the remaining 16 members contained the Pk_Tyr_Ser-Thr domain (Pfam ID: PF07714).

Therefore, candidate CrRLK1L members were characterized by the malectin-like domain (PF12819) in combination with either the Pk_Tyr_Ser-Thr domain (PF07714) or the Pkinase domain (PF00069). All candidate protein sequences were further verified using Pfam to confirm the presence of complete domain structures.

### Chromosomal distribution, phylogeny, and synteny analysis

Chromosomal loci were identified using genome annotation files in GFF format and visualized with TBtools [33]. Phylogenetic relationships were established using the Neighbor-Joining method in MEGA 11 under default parameters, following the alignment of amino acid sequences from *Arabidopsis* and the three *Brassica* species [34]. The online server iTOL (https://itol.embl.de/) was utilized to display and annotate the constructed tree. Additionally, to further investigate the evolutionary conservation and genomic synteny of CrRLK1L genes, collinearity analysis was conducted using MCScanX with default parameters [35].

### Gene structure, conserved motif, *cis*-elements analysis, and PPI network of *BnCrRLK1L* genes

The exon-intron organization of *BnCrRLK1L* genes was analyzed using the *B. napus* genome annotation files (GFF). Conserved motifs were identified via the MEME Suite server, while functional domains were examined using the NCBI Conserved Domain Database. The PlantCARE online tool was utilized to analyze the 2 kb genomic region upstream of the start codon for each *BnCrRLK1L* gene (http://bioinformatics.psb.ugent.be/webtools/plantcare/html) [36]. All the data were visualized using TBtools [33]. The PPI network of the CrRLK1L family in *B. napus* was constructed from STRING data [37] and visualized in Cytoscape [38]. Putative interacting genes were further evaluated through GO enrichment analysis using the BnaOmics platform [32].

### Transcriptomic profiling and qRT-PCR validation

Transcriptome data upon *S. sclerotiorum* inoculation were generated by RNA-seq in *B. napus* cv. ZS11 and ZY821 at 24 h post-inoculation in this study. Additionally, transcriptomic data covering 31 distinct tissues of the ZS11 cultivar were obtained from BnTIR (http://yanglaboratory.hzau.edu.cn/BnTIR) [39]. To maintain consistency with the data format retrieved from the BnTIR database, the expression levels of all analyzed genes were normalized and evaluated using TPM values. Expression profiles were visualized as heatmaps using TPM values through the ImageGP online server (https://www.bic.ac.cn/ImageGP/).

## Results

### Identification of *BnCrRLK1L1_5* as a candidate gene associated with resistance to *S. sclerotiorum*

**Fig. 1:**
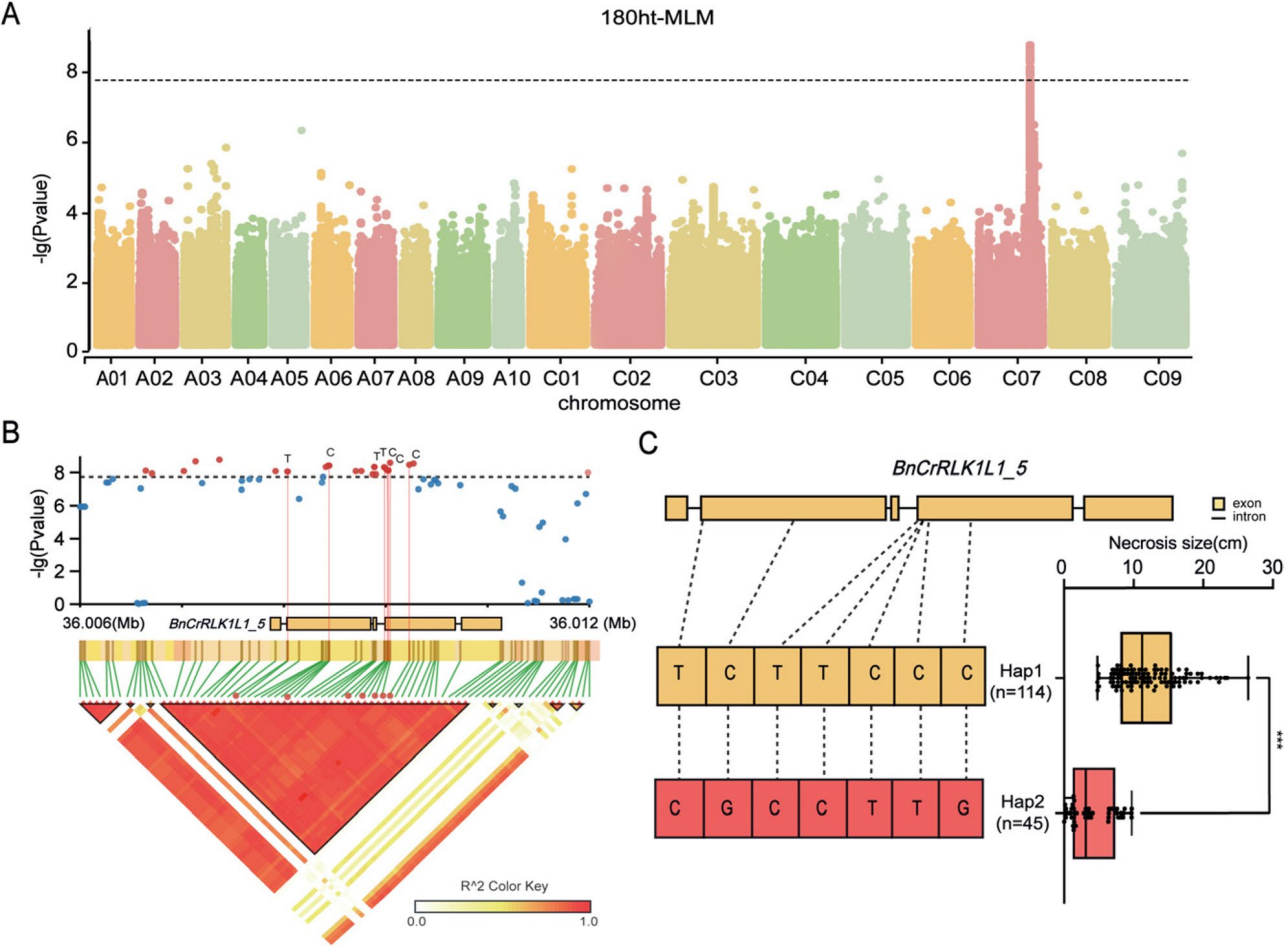
Discovery of the *S. sclerotiorum* resistance candidate gene *BnaC07g32280D* by GWAS. **A** Manhattan plot of the association analysis by MLM. Each point represents an SNP. The horizontal line indicates the genome-wide significance threshold (*p* = 1.787 × 10^–8^). **B** Location Manhattan plot and linkage disequilibrium (LD) heatmap of the candidate region (36.006–36.012 Mb) on C07. Red dots above the cutoff line represent significantly associated SNPs. **C** Structure and SNP variations of *BnaC07g32280D* and the necrosis size associated with the identified haplotypes. Data was analyzed via Student’s *t*-test (***, *p* < 0.001)

To investigate genes potentially regulating resistance to *S. sclerotiorum*, a GWAS was conducted using 180 *B. napus* accessions. A total of 2,797,642 SNPs with a MAF > 0.05 were analyzed using the MLM and GLM based on the average lesion size. Manhattan plots highlighted a genomic region significantly associated with resistance, spanning 36.006–36.012 Mb on chromosome C07 (Fig. 1A and Fig. S1). Within this 6-kb region, the most significant SNPs were in strong LD with BnaC07g32280D, which encodes a CrRLK1L family receptor-like kinase (Fig. 1B). Two major haplotype groups were identified by focusing on seven statistically significant missense SNPs in the candidate gene, comprising 45 (Hap1) and 114 (Hap2) accessions, respectively (Fig. 1C). Accessions in Hap2 exhibited significantly shorter lesion sizes than those in Hap1. The results suggest that *BnaC07g32280D* (a homolog of *AtCrRLK1L1*, also known as *FER*) is a strong candidate gene contributing to Sclerotinia stem rot resistance in *B. napus*.

### Identification and chromosomal distribution of CrRLK1L family in *B. napus*

To better characterize the gene identified by GWAS, a comprehensive genome-wide survey of the CrRLK1L family was performed in *B. napus*. Using 17 *Arabidopsis* CrRLK1L members as reference sequences, we identified 48 distinct CrRLK1L members in *B. napus*, including 23 in the A subgenome and 25 in the C subgenome (Table S3). These genes were unevenly distributed across 18 chromosomes, excluding A05 (Fig. S2 and Table S4). Chromosomes A04, C03, and C04 each carried five *CrRLK1L* genes, while A06 and C07 each carried four genes. The remaining chromosomes contained one to three *CrRLK1L* genes. These genes were named based on phylogenetic relationships with *Arabidopsis* orthologs. The gene *BnaC07g32280D* identified by GWAS in this study was an ortholog of *AtCrRLK1L1* (*FER*). Our results revealed six orthologous copies in *B. napus*, all showing high sequence similarity with *FER* (Fig. S3). Accordingly, *BnaC07g32280D* was named *BnCrRLK1L1_5.*

### Phenotype of *S. sclerotiorum* resistance and the expression profiling of *BnCrRLK1L1*

**Fig. 2:**
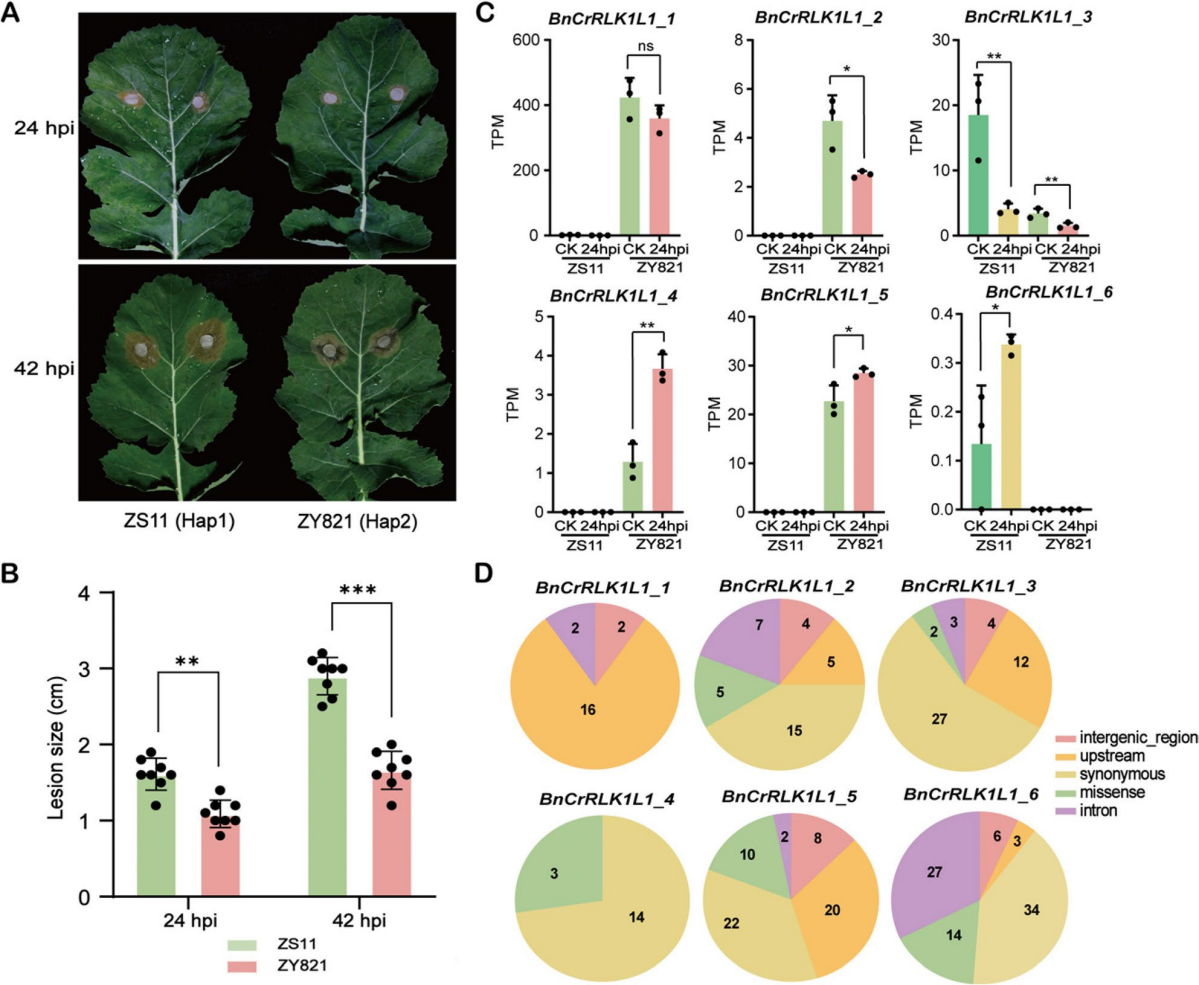
Phenotype and expression profiling of *BnCrRLK1L1* homologs in representative two haplotypes. **A** Disease symptoms on leaves of two haplotypes, including ZS11 (Hap1) and ZY821 (Hap2) at 24 and 42 h post-inoculation (hpi). **B** Quantification of lesion sizes at 24 and 42 hpi. **C** Expression patterns of *BnCrRLK1L1s* in ZS11 and ZY821 at 24 hpi. **D** Distribution of SNP variant types in the six *BnCrRLK1L1* gene loci. Different colored segments represent different SNP annotation categories. Data was analyzed via Student’s *t*-test: ns, no significance; *, *p* < 0.05; **, *p* < 0.01; ***, *p* < 0.001

Representative accessions representing the two haplotypes were subjected to leaf inoculation assays. The resistant genotype (Hap2) exhibited significantly delayed symptom development compared with the susceptible genotype (Hap1) at both 24 and 42 h post-inoculation (hpi) (Fig. 2A). Quantification of lesion size confirmed that Hap2 accessions developed significantly smaller necrotic lesions than Hap1 (Fig. 2B), validating the association with Hap2 and the enhanced *S. sclerotiorum* resistance.

To identify the causal gene among the homologs, the expression patterns of the six *BnCrRLK1L1* copies were examined upon *S. sclerotiorum* inoculation. Of these, four genes were differentially expressed between the two haplotypes. *BnCrRLK1L1_4* and *BnCrRLK1L1_5* shared similar expression patterns. Their transcripts were barely detectable in Hap1 but were strongly and rapidly induced upon inoculation in Hap2 (Fig. 2C). Furthermore, analysis of sequence variations within the genomic regions of the six *BnCrRLK1L1* genes revealed that *BnCrRLK1L1_5* (62 SNPs) and *BnCrRLK1L1_6* (84 SNPs) exhibited a higher degree of overall polymorphism than the other homologs (Fig. 2D). Notably, *BnCrRLK1L1_5* harbored the highest number of upstream regulatory variants (20 SNPs) and a large number of missense mutations (10 SNPs), suggesting potential functional variation (Fig. 2D). Combined with its inducible expression pattern, these features implicate BnCrRLK1L1_5 as the causal candidate gene.

### *BnCrRLK1L1_5* enhances resistance to stem rot and inhibits BAX-induced cell death

**Fig. 3:**
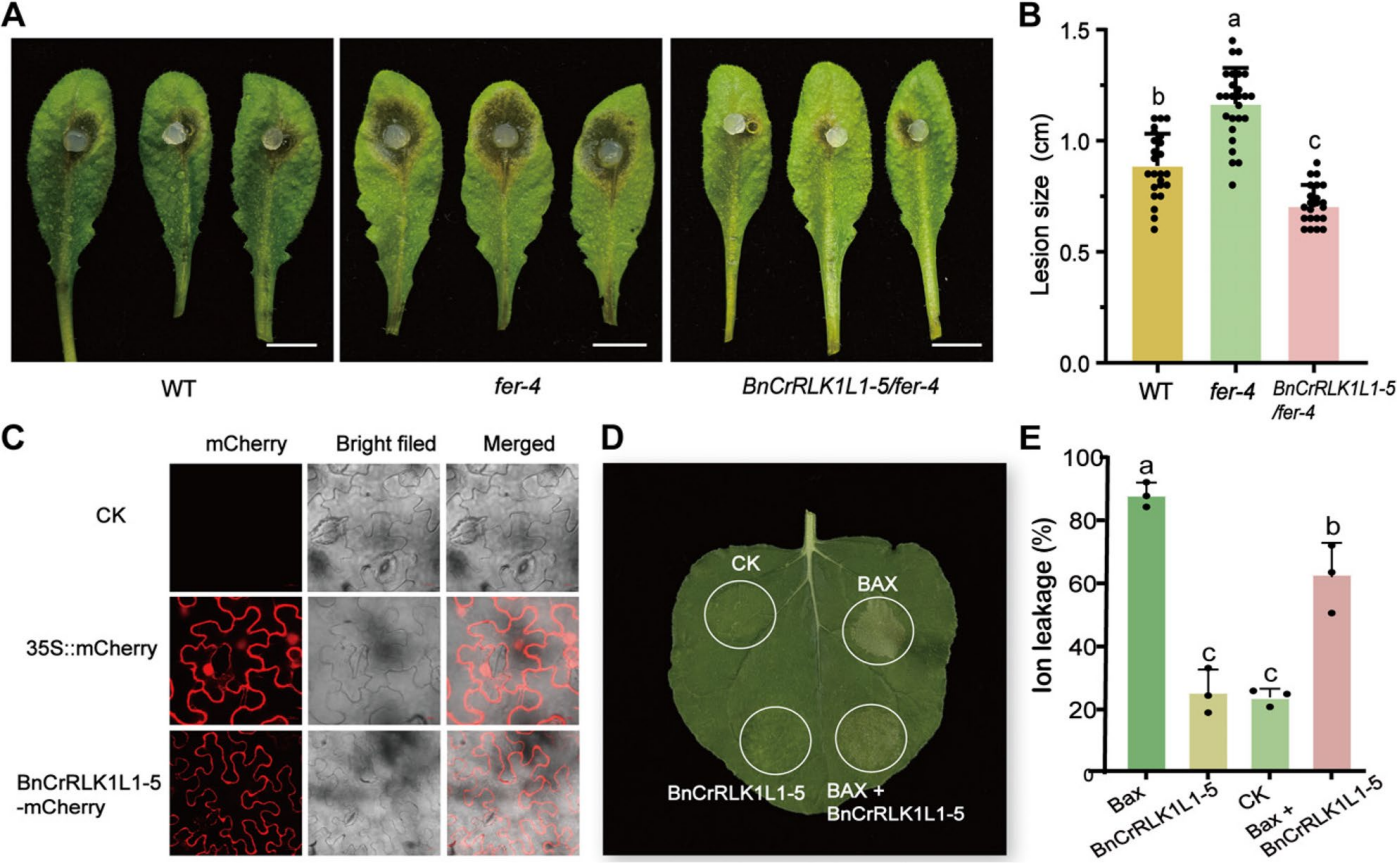
*BnCrRLK1L1_5* enhances resistance to *S. sclerotiorum* and suppresses BAX-induced cell death.** A** Phenotype of Arabidopsis WT, *fer-4* mutant, and *BnCrRLK1L1_5* complementation line at 48 h post-inoculation (hpi). **B** Statistical analysis of lesion sizes at 48 hpi. **C** Subcellular localization of *BnCrRLK1L1_5*-mCherry fusion protein in *Nicotiana benthamiana*. **D** Leaf necrosis identification at 5 days post-infiltration (dpi) using a transient expression system in *N. benthamiana.* **E** Measurement of ion leakage at 3 dpi in *N. benthamiana*. Distinct letters denote statistically significant differences (*p* < 0.05)

Since *BnCrRLK1L1_5* is an ortholog of the Arabidopsis receptor kinase *FERONIA (FER)*, the loss-of-function mutant *fer-4* was inoculated with *S. sclerotiorum* to assess resistance. The *fer-4* mutant displayed significantly expanded lesions compared with the wild-type upon inoculation with *S. sclerotiorum* (Fig. S4 and Fig. 3A-B), indicating that *FER* is putatively required for basal resistance against this pathogen. A construct carrying the full gene sequence of *BnCrRLK1L1_5* (haplotype from ZY821) with its native promoter was transformed into the *fer-4* mutant for complementation (Fig. S5 and Fig. S6). The transgenic plants exhibited significantly reduced disease symptoms and restored resistance to levels exceeding the wild-type, indicating that *BnCrRLK1L1_5* can compensate for the loss of resistance in the *fer-4* mutant (Fig. 3A-B). These results support a pivotal role for *BnCrRLK1L1_5* in positively regulating immunity against *S. sclerotiorum*.

Subcellular localization studies in *N. benthamiana* leaves showed that BnCrRLK1L1_5-mCherry fusion protein localized on the cell membrane (Fig. 3C). Furthermore, the co-expression of *BnCrRLK1L1_5* with BAX significantly alleviated leaf necrosis, indicating that *BnCrRLK1L1_5* inhibits BAX-triggered cell death (Fig. 3D). The rate of ion leakage was also significantly reduced in co-expressed *N. benthamiana* leaves, further supporting the result that *BnCrRLK1L1_5* inhibited cell death (Fig. 3E). The results suggest that *BnCrRLK1L1_5* contributes to resistance, likely by suppressing pathogen-associated cell death.

### Phylogenetic and syntenic relationship of the CrRLK1L family

**Fig. 4:**
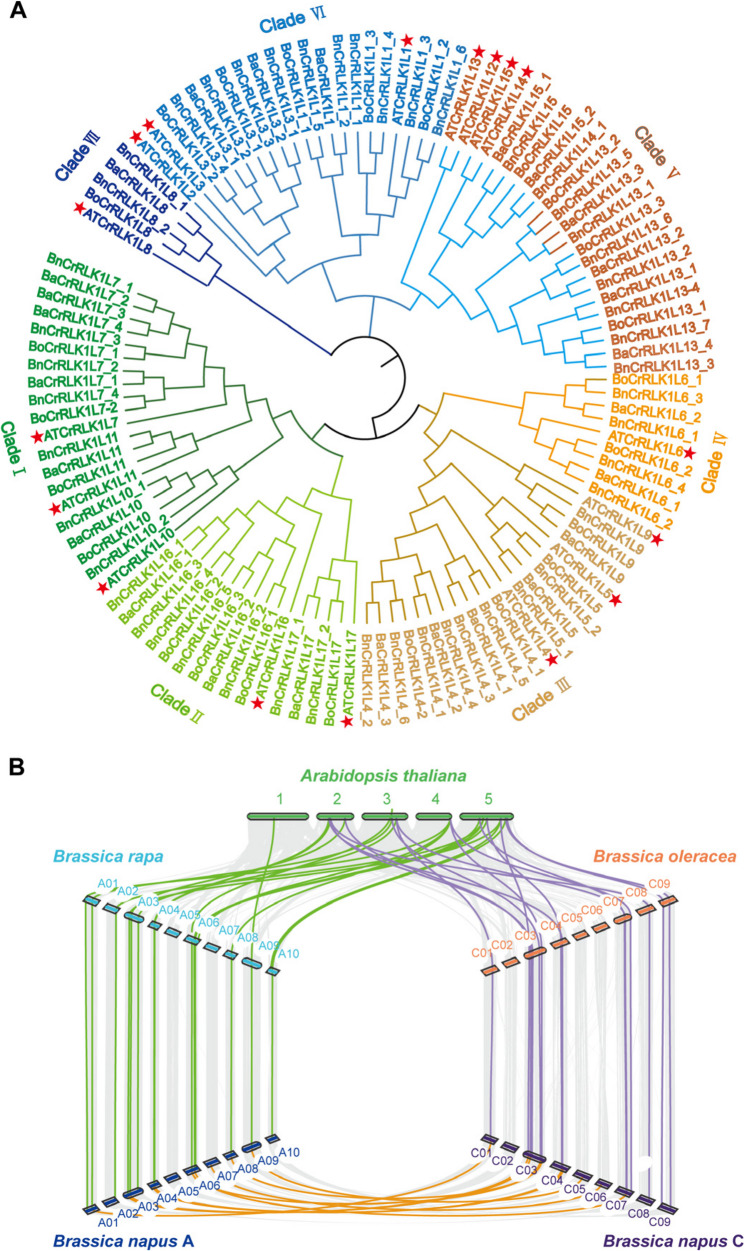
Phylogenetic and syntenic analyses of the CrRLK1L family in *Arabidopsis* and *Brassica* species. **A** Phylogenetic tree generated with MEGA 11, showing seven distinct clades (indicated by different colors). **B** Syntenic relationships of the CrRLK1L family indicate the syntenic gene pairs within *Arabidopsis* and *Brassica* species. Gray lines denote syntenic blocks. Colored lines trace specific syntenic relationships: green for *(A) thaliana*, *(B) rapa*, and the *B. napus* A subgenome; purple for *(A) thaliana*, *(B) oleracea*, and the *B. napus* C subgenome; and orange for pairs between the A and C subgenomes of *B. napus*

To explore the evolutionary relationship within the CrRLK1L family, an unrooted phylogenetic tree was generated from 114 amino acid sequences, including 17 CrRLK1L proteins from *(A) thaliana*, 25 from *(B) rapa*, 24 from *B. oleracea*, and 48 from *B. napus* (Table S5). These proteins were classified into seven distinct clades based on phylogenetic relationships (Fig. 4A). Clade V, with 23 members, was identified as the largest cluster, containing *CrRLK1L12* to *15*. Clade III and VI each contained 21 members, including CrRLK1L4/5/9 and CrRLK1L1/2/3, respectively. Most *AtCrRLK1L* genes had 2–6 orthologs in *B. napus*, distributed across both the A and C subgenomes. This expansion reflects the whole-genome duplication events in *Brassica*. However, specific evolutionary divergence was observed. For example, *AtRLK1L9/11/14/15* each had only one ortholog, whereas *AtRLK1L13* had seven orthologs. The phylogenetic analysis also revealed the loss of *AtCrRLK1L2/12* orthologs in *Brassica* species, suggesting gene loss events during evolution. Within clade VI, the candidate gene *BnCrRLK1L1_5* and its paralog *BnCrRLK1L1_3* both clustered tightly with *AtCrRLK1L1*. While *BnCrRLK1L1_3* showed slightly higher sequence similarity, the close phylogenetic relationship of *BnCrRLK1L1_5* within this specific subclade suggested a shared evolutionary lineage and potential functional conservation.

Syntenic analysis highlighted the conservation of *CrRLK1L* genes between *Arabidopsis* and the *Brassica* crops (Fig. 4B and Table S5). However, the synteny between *(A) thaliana* and *Brassica* genomes appeared fragmented, reflecting extensive chromosomal rearrangements during the evolution of *Brassica* species. Furthermore, a one-to-one syntenic relationship was revealed between the *(B) napus* A subgenome and *B. rapa* chromosomes, and between the *B. napus* C subgenome and *B. oleracea* chromosomes, confirming the allopolyploid origin and genomic stability in *B. napus*.

### Analyses of gene structure, conserved domain, and protein motif in BnCrRLK1L proteins

**Fig. 5:**
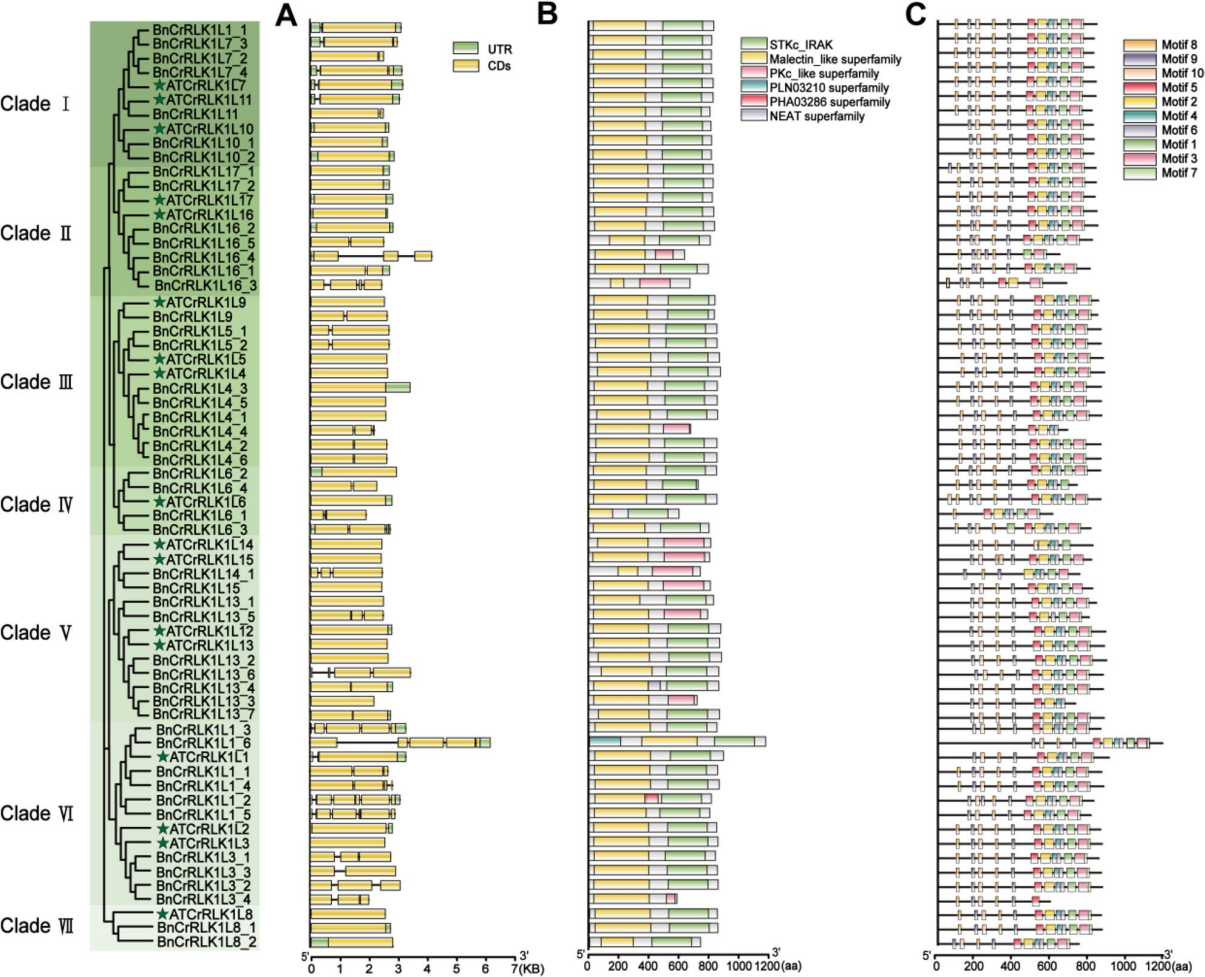
Phylogenetic relationships and structural characterization of CrRLK1L members in *B. napus* and *Arabidopsis.* A phylogenetic tree is generated based on the sequences of AtCrRLK1L and BnCrRLK1L. **A** Exon-intron organization. The UTRs and exons are represented by light green and light yellow boxes, respectively, while introns are represented by black lines. **B** Functional domains. Malectin-like (PF12819) and malectin (PF11721) domains are shown in red and pink, respectively; kinase domains including Pkinase (PF00069) and STKc-IRAK (PF07714) are marked in yellow. Corresponding Pfam accessions are listed in Table S3. **C** Distribution of conserved motifs, with each motif represented by a distinct color. Detailed motif sequences are provided in Table S6

To explore the structural diversity of the BnCrRLK1L proteins, we examined the exon-intron organization, conserved domains, and protein motifs of the 48 BnCrRLK1L proteins (Fig. 5). Gene structure analysis revealed that almost half of the family members lacked introns, and about one-third contained a single intron. All CrRLK1L family members feature conserved malectin-like and protein kinase domains. Conserved domain analysis confirmed that all BnCrRLK1L proteins share the standard structure of receptor-like kinases. Specifically, they feature tandem N-terminal malectin (PF11721) or malectin-like (PF12819) domains, followed by a transmembrane helix, and C-terminal kinase domain including Pkinase (PF00069) and Pk_Tyr_Ser-Thr (PF07714). Analysis of conserved motifs in the BnCrRLK1L proteins revealed 9–10 motifs across the family, with some motifs repeated in certain genes and other motifs absent in specific members (Table S6). For example, motifs 3 and 7 were absent in some genes, while motif 8 was absent in the group V members.

### Spatiotemporal expression pattern of *BnCrRLK1L* genes

**Fig. 6:**
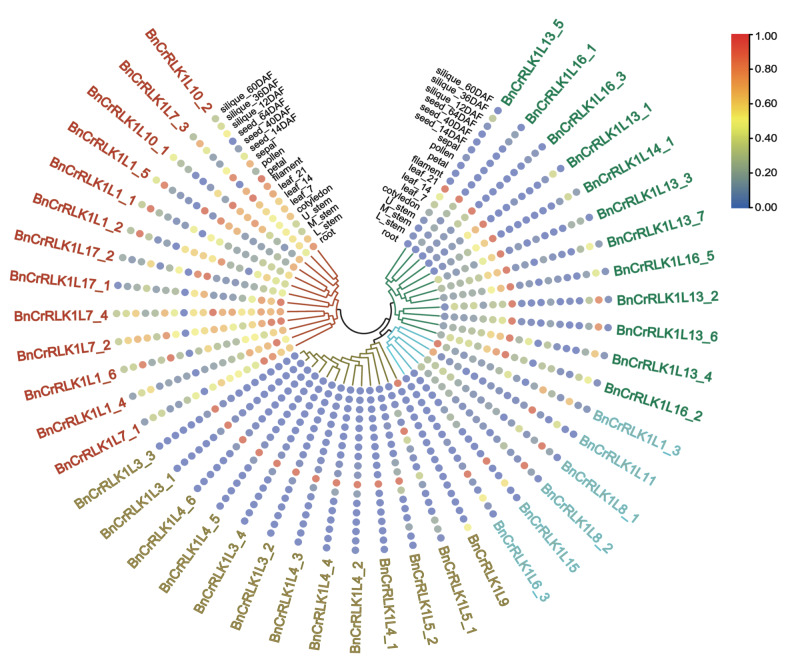
Tissue-specific expression profiles of the *BnCrRLK1L* gene family. The heatmap displays the expression levels (log_2_-transformed TPM values) of *BnCrRLK1L* genes. The four phylogenetic clades are distinguished by different colors. The tested tissues include root, L_stem (lower_stem_peel), M_stem (middle_stem_peel), U_stem (upper_stem_peel), cotyledon, leaf (7, 14, and 41 days old), filament, petal, pollen, sepal, seed (14, 40, and 64 days after flowering), and silique (12, 36, and 60 days after flowering). Relative transcript abundance is indicated by the color scale, ranging from blue (low) to red (high)

Based on RNA-seq data from 18 tissues at different developmental stages, *BnCrRLK1L* genes exhibiting distinct expression profiles were classified into four clades (Fig. 6). The green clade, including *BnCrRLK1L13*/*14*/*16*, showed predominantly expression in leaves, and the genes in the blue clade were highly expressed in seeds and siliques. The gold clade, including *BnCrRLK1L3*/*4*/*5*, displayed a pollen-specific expression. The red clade, including *BnCrRLK1L1*/*7*/*17*, exhibited ubiquitous expression across most tissues. Within this clade, *BnCrRLK1L1* genes showed varied expression patterns. The candidate *BnCrRLK1L1_5* exhibited high expression levels in most tissues but relatively low transcript levels in seeds, pollen, and stems. Overall, the similar expression patterns of different *BnCrRLK1L* paralogs suggest conserved functional roles across the gene family.

### *Cis*-elements analysis of *BnCrRLK1L* genes

**Fig. 7:**
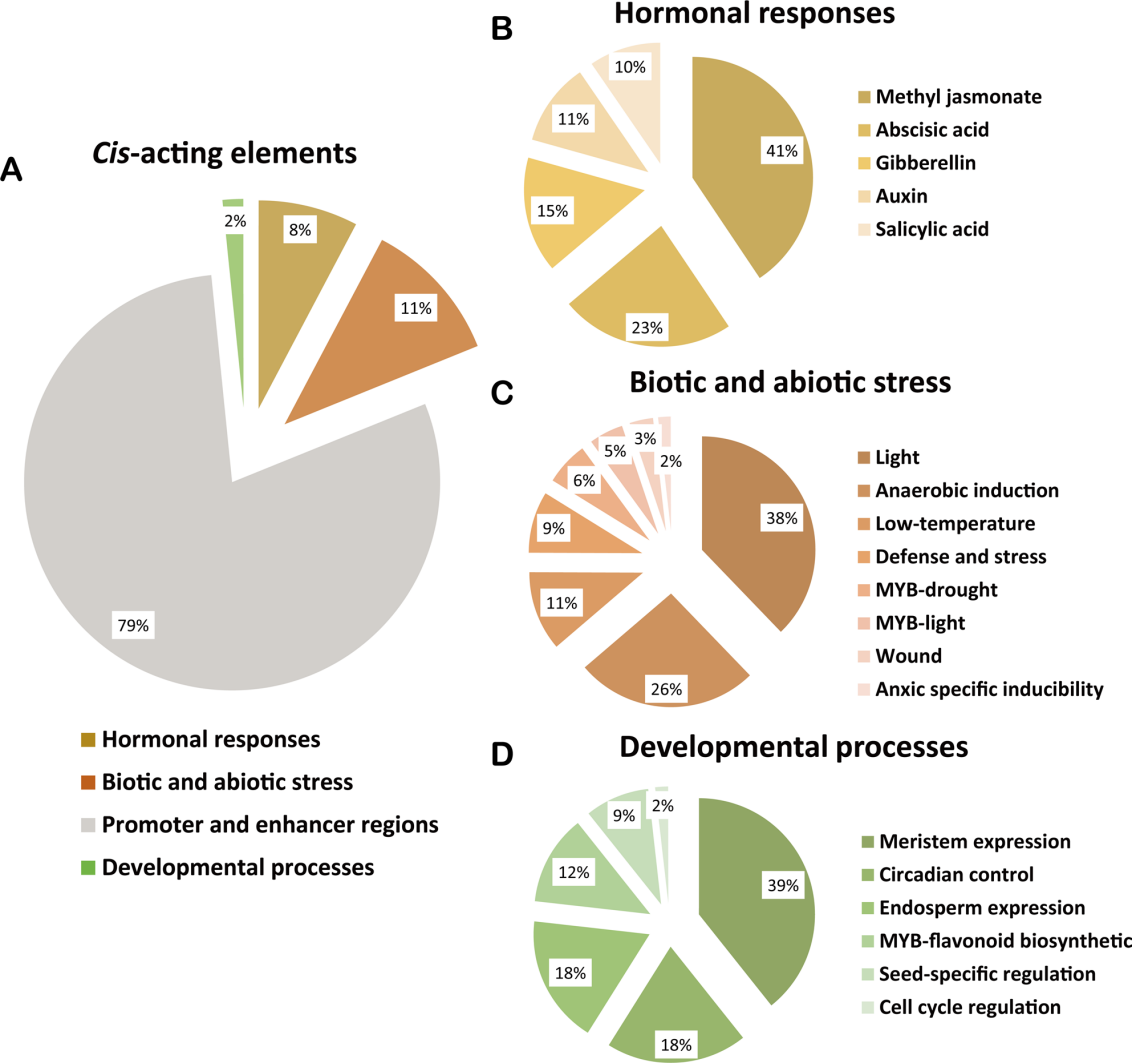
Distribution and functional classification of *cis*-elements in *BnCrRLK1L* promoters. **A** Overall distribution of all identified *cis*-elements. **B**-**D** Number and categorization of *cis*-elements associated with hormone responses (**B**), biotic and abiotic stress responses (**C**), and developmental processes (**D**)

To understand the transcriptional regulation mechanisms of *BnCrRLK1L* genes, *cis*-regulatory elements were analyzed in the promoters (Table S7). The analysis showed that core promoter and enhancer region elements accounted for approximately 79% of all predicted *cis*-elements (Fig. 7A and Fig. S7). The remaining elements were mainly associated with hormonal response, stress, and developmental processes. About 8% of hormone-responsive *cis*-elements were enriched. Among them, MeJA-responsive elements were the most significantly enriched, followed by ABA-responsive elements (Fig. 7B). The enrichment of MeJA-responsive elements reflects an evolutionary adaptation of these genes for defense against necrotrophic pathogens. Moreover, multiple environmental stress-responsive elements were also identified, including light, anaerobic conditions, drought, low temperature, defense, and wounding (Fig. 7C). This indicates that the expression of *BnCrRLK1L* is putatively regulated by environmental stimuli. In addition, a smaller proportion of *cis*-elements was associated with various developmental processes, including meristem activity, circadian regulation, endosperm expression, flavonoid biosynthesis, seed regulation, and cell cycle control (Fig. 7D), indicating the diverse roles in development.

### PPI network of BnCrRLK1L members

**Fig. 8:**
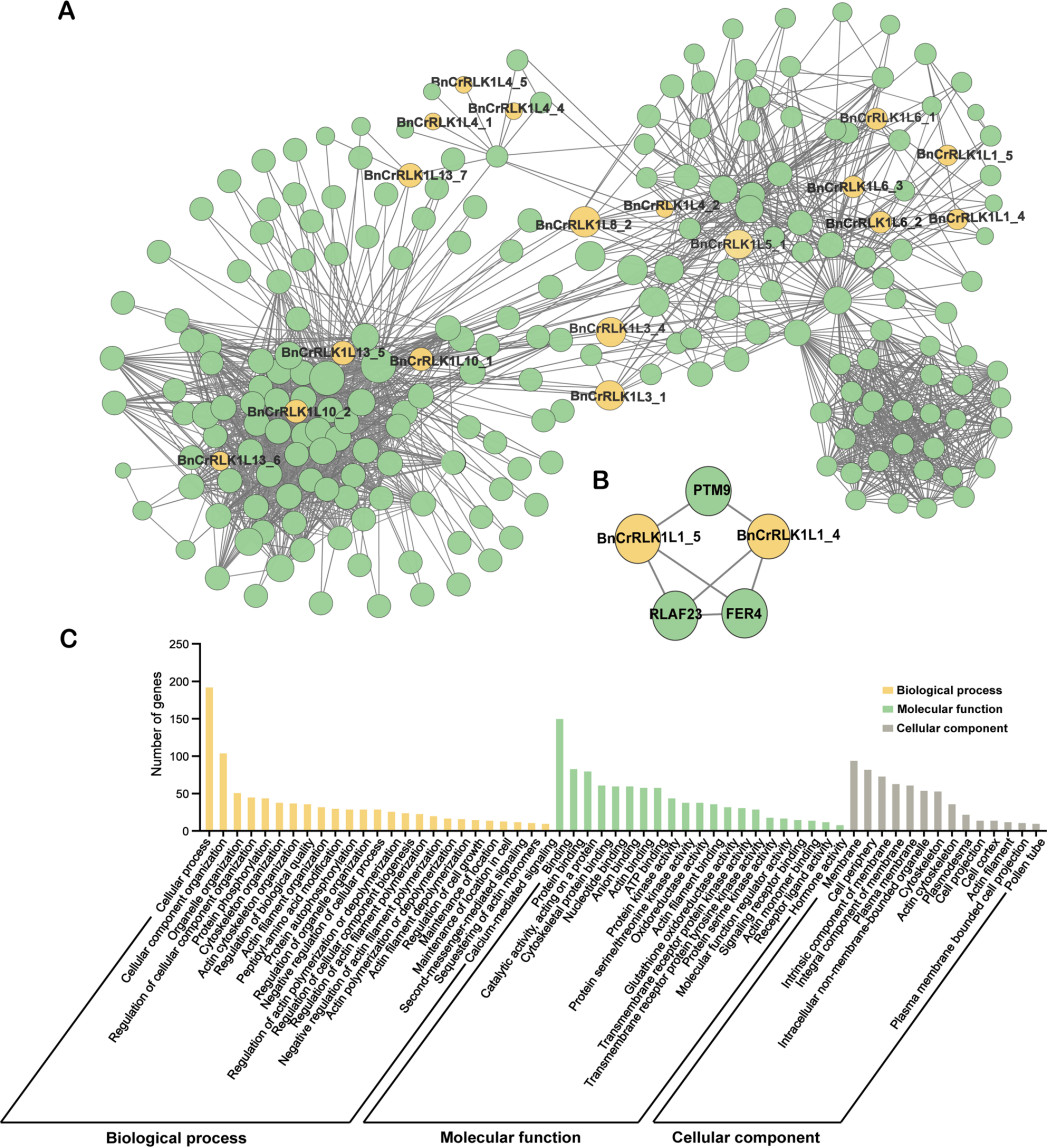
Prediction of PPI network for BnCrRLK1L members. **A** Global PPI network constructed using STRING. The yellow nodes represent the query BnCrRLK1L protein, and the green nodes indicate interacting proteins. The gray lines represent predicted interactions. **B **A specific subnetwork for BnCrRLK1L1_5. PTM9, protein methyltransferases 9; RALF23, RAPID ALKALINIZATION FACTOR 23; FER4, ferritin 4. **C** GO classification of the potential interacting proteins

To explore potential interaction partners, a PPI network for BnCrRLK1L members was generated using the STRING database (Fig. 8A and Table S8). The resulting network of *B. napus* comprised 190 nodes and 930 edges, with two major clusters suggesting divergent signaling roles within the family. Among the 190 nodes, 14 represented query BnCrRLK1L proteins, which act as hubs connecting to numerous interacting partners.

A focused subnetwork concentrated on BnCrRLK1L1_5 was identified (Fig. 8B). This subnetwork contained crucial nodes, including its paralog BnCrRLK1L1_4, the peptide ligand RALF23 (Rapid Alkalinization Factor 23), PTM9 (Protein Methyltransferase 9), and FER4 (Ferritin 4). BnCrRLK1L1_4 not only physically interacts with BnCrRLK1L1_5 but also shares a highly similar expression pattern (Fig. 2C), indicating that they may cooperate in *S. sclerotiorum* resistance. However, the possibility of functional redundancy cannot be ruled out. Moreover, the receptor-like protein RALF23 was also identified in this subnetwork, implicating its conserved role in RALF-triggered immunity.

GO enrichment analysis of the interaction partners provided insights into the cellular mechanisms of BnCrRLK1L proteins. Functional annotation based on GO enrichment indicated that the interacting proteins were significantly associated with biological processes, molecular functions, and cellular components (Fig. 8C and Table S9). Biological process terms were primarily associated with cellular processes such as the organization of cellular components, cytoskeleton dynamics, and actin filament assembly. Analysis of molecular function revealed a significant enrichment in binding-related activities, particularly cytoskeletal protein binding, nucleotide binding, and ATP binding. For cellular components, the analysis reveals a significant enrichment in membrane-related categories, including the cell periphery and integral membrane components. These results suggest that BnCrRLK1L proteins may be important for cell membrane integrity and intracellular signaling, processes closely linked to plant defense responses.

## Discussion

### *BnCrRLK1L1_5* enhances resistance to *S. sclerotiorum* in *B. napus*

Our study highlights the significance of the *CrRLK1L* receptor-like kinase family in crop immunity. *FER* has been widely reported to regulate broad-spectrum immunity in the model plant *Arabidopsis* [1]. However, understanding its homologous functions in polyploid crops like *B. napus* is challenging due to extensive gene family expansion and functional redundancy [17].

Although Hap1 is broadly distributed across susceptible accessions, the highly resistant Hap2 allele is relatively rare. The strong pathogen-induced transcriptional activation of Hap2 suggests an efficient and rapid immune response mechanism. The low frequency of this advantageous allele in the examined population indicates that it has largely escaped extensive selection during the historical domestication of rapeseed [39]. However, large-scale haplotype identification and association analysis in germplasm resources are necessary to evaluate the application potential of Hap2 in breeding populations and to develop molecular markers for assisted selection.

Furthermore, the biological significance of *BnCrRLK1L1_5* is strongly reinforced by its functional conservation. Previous studies have established that *FER* acts as a crucial positive regulator against necrotrophs, including *S. sclerotiorum* and *Botrytis cinerea* [40]. Our heterologous expression data demonstrated that *BnCrRLK1L1_5* not only fully rescued the susceptibility of the *Arabidopsis fer-4* mutant but also conferred resistance levels surpassing those of the wild-type. The enhanced resistance may result from the potent immune signaling of the naturally selected Hap2 allele in *B. napus*. Additionally, it is also possible that sequence divergence protects *BnCrRLK1L1_5* from the *S. sclerotiorum* effectors that normally hijack the native *Arabidopsis FER* [41]. While its role in conferring basal resistance is clear, future comparative studies are required to elucidate how specific sequence variations between Hap1 and Hap2 alter this protective kinase function.

### Potential mechanism of *BnCrRLK1L1_5*-mediated defense against *S. sclerotiorum*

Cell death plays a versatile role during the interplay between plants and pathogens. On one hand, cell death is part of the plant’s defense strategy to restrict pathogen spread and to confer resistance against biotrophic pathogens [42]. However, necrotrophic pathogens such as *S. sclerotiorum* exploit cell death to facilitate infection and colonization by secreting effectors that degrade plant cell walls and induce additional cell death [43, 44]. Accordingly, suppression of excessive cell death represents an effective resistance strategy for necrotrophic pathogens [45]. In this study, co-expression of *BnCrRLK1L1_5* with *BAX* in *N. benthamiana* leaves significantly reduced necrosis and ion leakage, suggesting that *BnCrRLK1L1_5* functions as a suppressor of cell death.

Previous studies have shown that suppression of necrotic cell death is a known strategy confering resistance to *S. sclerotiorum*. Overexpression of *AtMLO2* and *BnMLO2_2* in *Arabidopsis* reduced lesion sizes and cell death following *S. sclerotiorum* infection [46]. Similarly, *FER* has been demonstrated to modulate immune responses through RALF-mediated signaling, and *fer* loss-of-function mutants exhibit enhanced cell death and altered defense responses [3, 47]. A recent study demonstrated that RALF22, a peptide involved in cell wall remodeling, inhibits ROS bursts, thereby positively regulating the resistance to *S. sclerotiorum* [40]. Together, the results suggest that the resistance conferred by *BnCrRLK1L1_5* is achieved by suppressing necrotic cell death.

PPI network analysis further provided insights into the potential molecular mechanisms. BnCrRLK1L1_5 was found to interact with several important proteins. RALF23 is a well-characterized FER ligand that regulates immune signaling and cell wall remodeling [13], suggesting that BnCrRLK1L1_5 may participate in peptide-mediated signaling at the cell surface. Ferritin 4, which is involved in ion homeostasis, may regulate oxidative stress during pathogen attack [48]. By promoting Ferritin stability or accumulation, *BnCrRLK1L1_5* could alleviate cell death, thereby restricting the infection of the necrotrophic pathogen *S. sclerotiorum*. Moreover, *BnCrRLK1L1_4* shared similar expression patterns with *BnCrRLK1L1_5* upon *S. sclerotiorum* inoculation, raising the possibility that these two genes act together or redundantly to modulate resistance to *S. sclerotiorum.* These interactions support a model about how *BnCrRLK1L1_5* may contribute to resistance by modulating cell wall integrity, oxidative stress, and immune signaling.

### Evolutionary dynamics and functional redundancy of the CrRLK1L family

Despite the extensive characterization of the CrRLK1L family in various plant species, a systematic genome-wide analysis in *B. napus* remains lacking [49–55]. As an allopolyploid species derived from hybridization between *B. rapa* and *B. oleracea*, *B. napus* provides an opportunity to investigate gene family expansion and functional divergence following polyploidization [17, 56]. Phylogenetic and synteny analyses suggest that the CrRLK1L family has undergone extensive expansion and diversification across *Brassica* species. Although the total number of *CrRLK1L* genes in *B. napus* approximates the combined numbers found in its progenitor species, differential retention of orthologs between the A and C subgenomes indicates asymmetric gene loss and chromosomal rearrangement following polyploidization [57].

Despite diversification, the CrRLK1L family exhibited significant evolutionary conservation, particularly within key functional domains, including the malectin-like and kinase domains [58]. The functional complementation of Arabidopsis *fer-4* by *BnCrRLK1L1_5* in this study provides experimental evidence for the conservation of CrRLK1L1 function across species. Moreover, the presence of six copies of the *BnCrRLK1L1* genes in *B. napus* suggests specialized roles in different tissues or developmental stages. Interestingly, *BnCrRLK1L1_5* and *BnCrRLK1L1_4* not only share high sequence identity with *FER* but also exhibit co-expression patterns and potentially interact with each other. The findings support the hypothesis that these paralogs may act cooperatively or redundantly in conferring resistance to *S. sclerotiorum.* Such functional redundancy may enhance the robustness of defense responses against necrotrophic pathogens and contribute to adaptive evolution of disease resistance in polyploid crops [59].

## Conclusion

We identified *BnCrRLK1L1_5* as a key positive regulator of resistance to *S. sclerotiorum*, potentially acting through suppression of host cell death. The study highlights the potential for manipulating the CrRLK1L family to improve disease resistance in *Brassica* crops. Further studies are needed to elucidate the exact molecular function of *BnCrRLK1L1_5* and its homologous genes, especially *BnCrRLK1L1_4*, which regulates defense response to *S. sclerotiorum* inoculation in *B. napus*.

## Supplementary Information

Supplementary Material 1.

Supplementary Material 2.

## Data Availability

The datasets generated and/or analysed during the current study are available on request.

## Abbreviations

GWAS: Genome-wide association studies
RLK: Receptor-like kinase
RALF: Rapid alkalinization factor
ROS: Reactive oxygen species
PRRs: Ppattern recognition receptors
SNP: Single nucleotide polymorphism
PDA: Potato dextrose agar
MAF: Minimum allele frequency
MLM: Mixed linear model
GLM: General linear model
GFF: General feature format
PPI: Protein-protein interaction
GO: Gene ontology
TPM: Transcripts per Million
