# Supplementary material for: Genome-wide association study and evolutionary analysis of the CrRLK1L family reveal BnCrRLK1L1_5 as a positive regulator of Sclerotinia sclerotiorum resistance in Brassica napus
Source: BMC Plant Biol. 2026 May 8;26:1108. doi: 10.1186/s12870-026-08872-5 (PMC13321965; doi:10.1186/s12870-026-08872-5)
Supplement: Supplementary file 1 — Supplementary Material 1. [file 12870_2026_8872_MOESM1_ESM.doc]

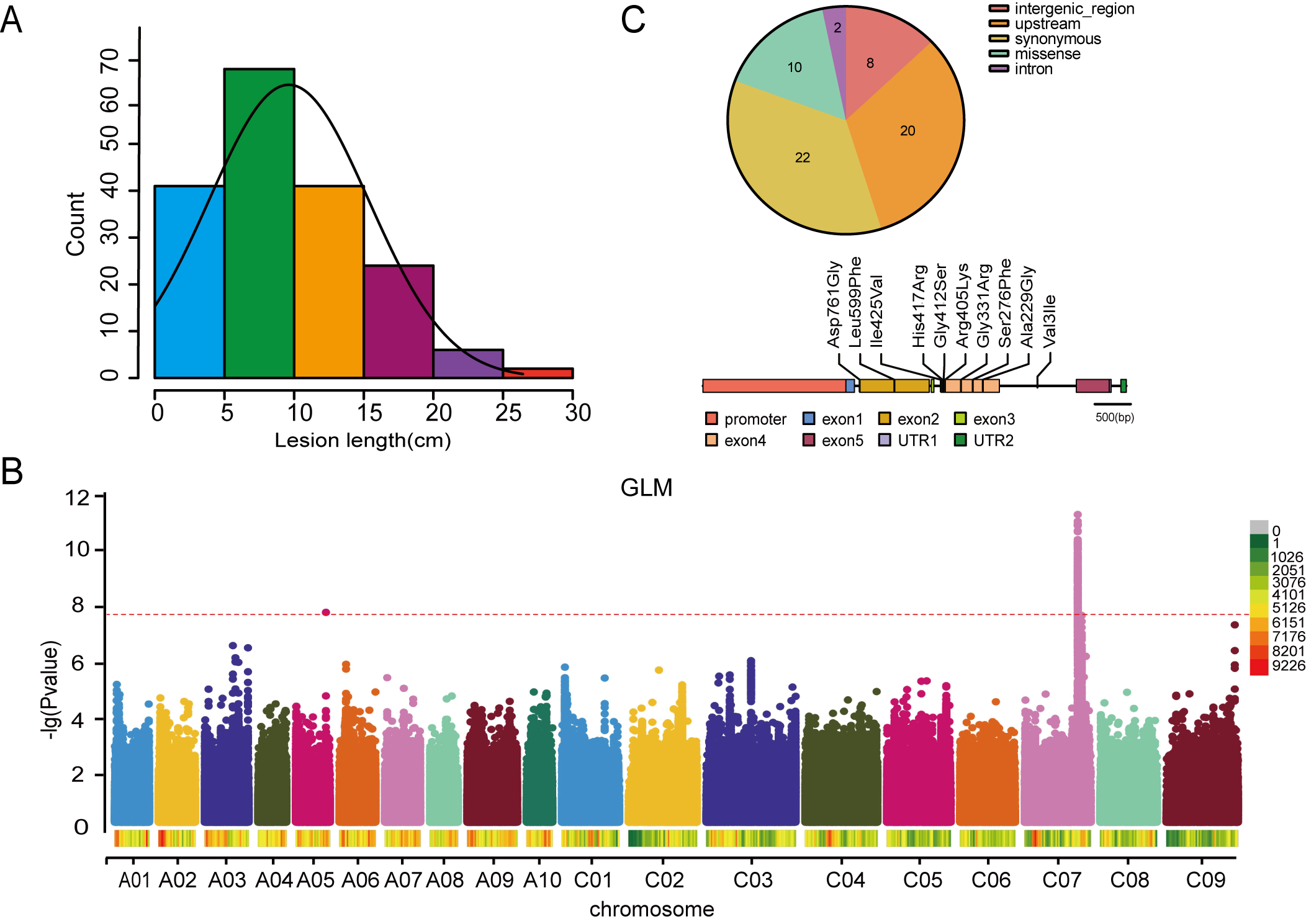


**Fig. S1** **Phenotypic distribution, GWAS results, and SNP variation analysis.** (A) Phenotypic distribution of stem inoculation with *S. sclerotiorum* in natural popoulation. (B) Manhattan plot of the disease index from association analyses by General Linear Model (GLM). (C) Distribution of SNP variant types in *BnaC07g32280D* gene loci.

**
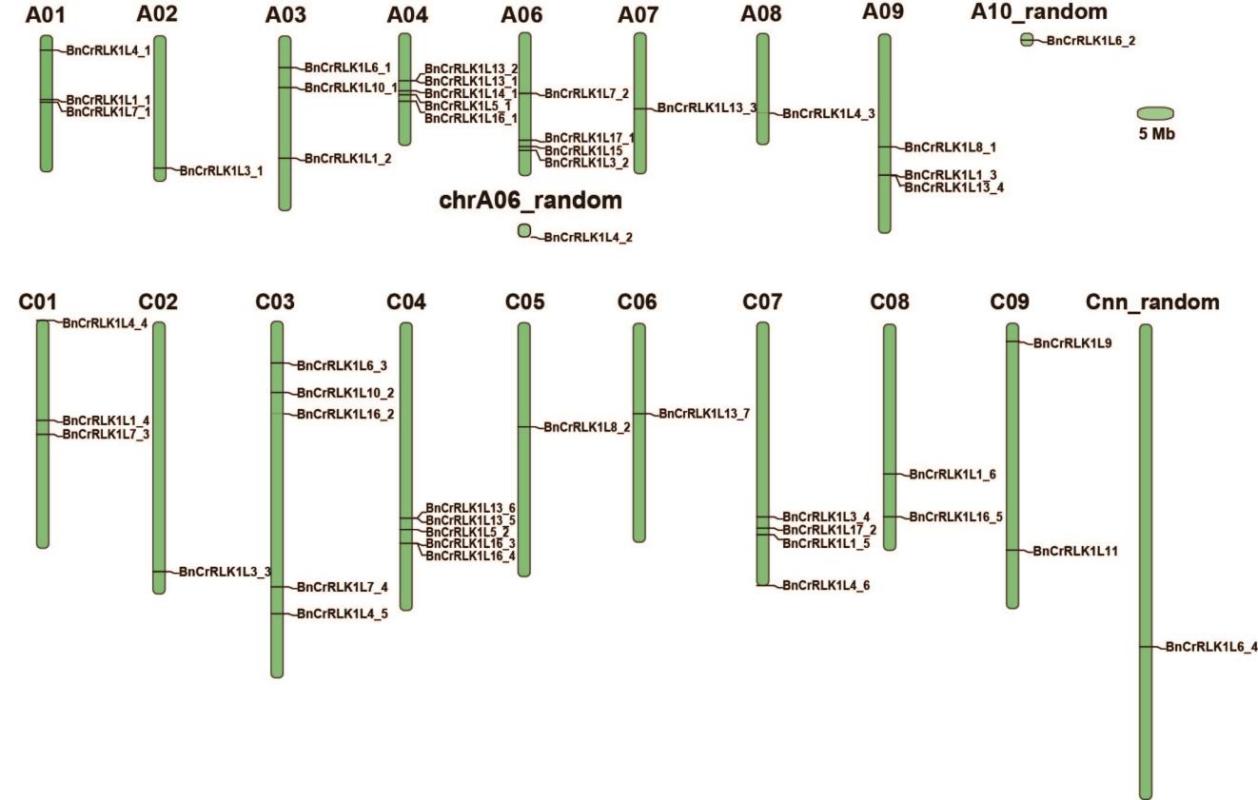
**

**Fig. S2 Chromosomal distribution of CrRLK1L genes in B. napus.**


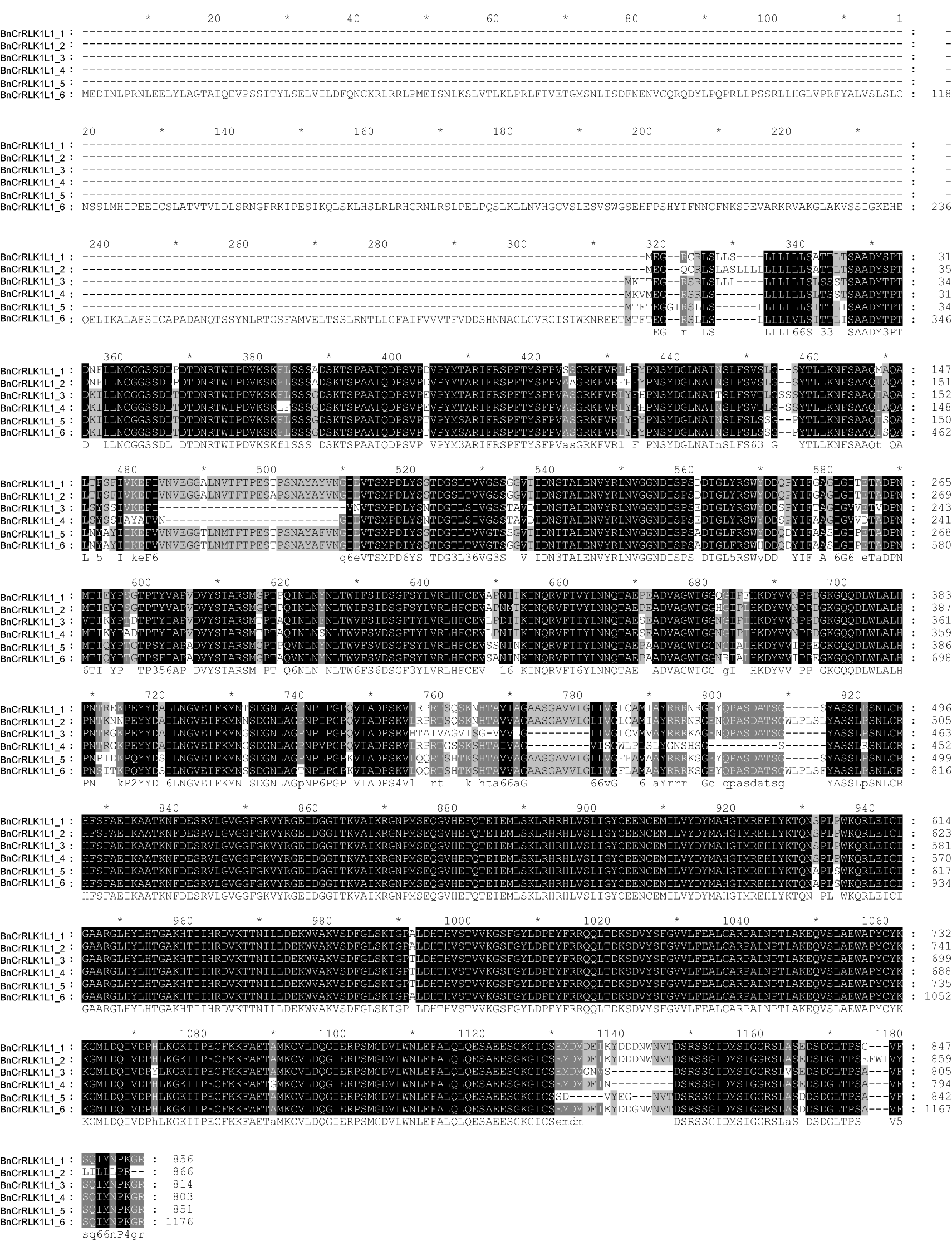


**Fig. S3 Sequence alignment of** **BnCrRLK1L1 identified in *B. napus* Darmor-bzh reference genome.** Identical sequences are highlighted with black backgrounds within the six BnCrRLK1L1 proteins.


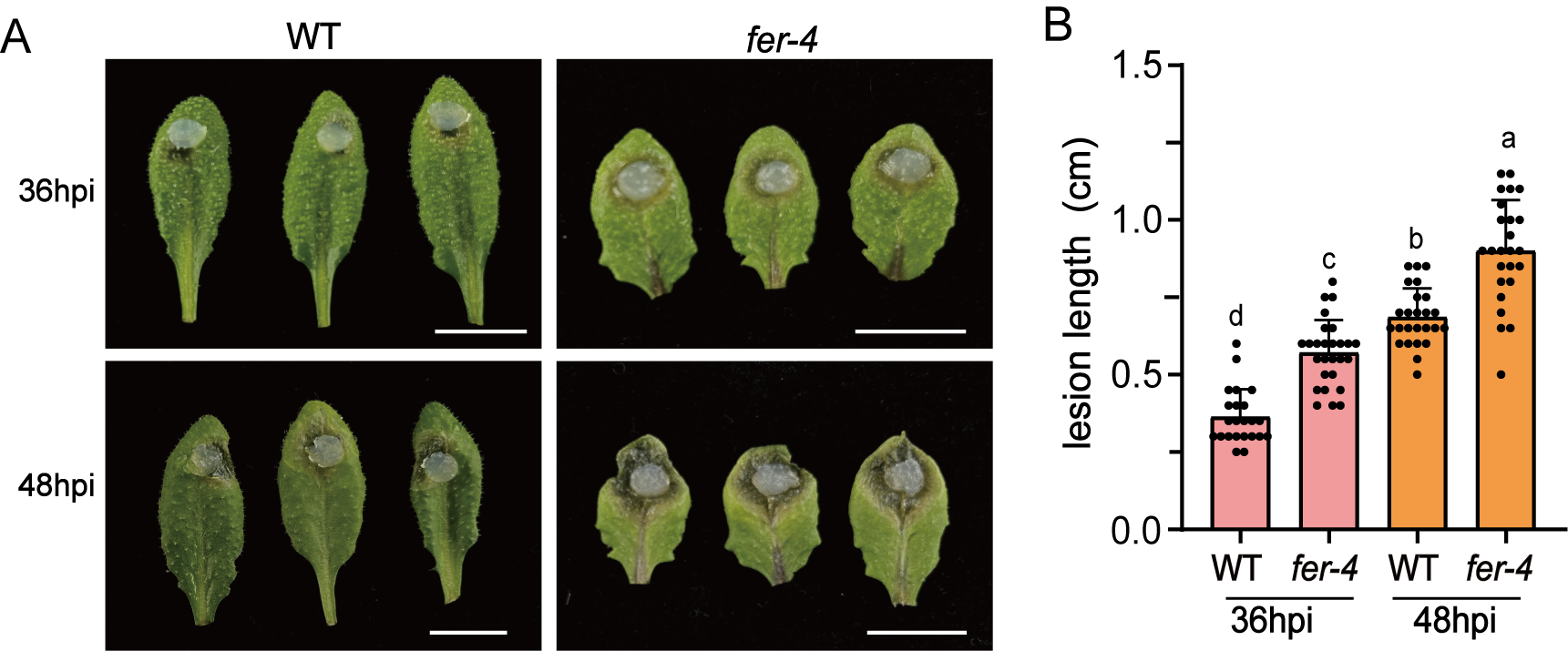


**Fig. S4 Susceptibility assessment of the *Arabidopsis fer-4* mutant to *S. sclerotiorum*.** (A) Representative disease symptoms on leaves of wild-type (WT) and *fer-4* mutant plants at 36 and 48 hours post-inoculation (hpi). (B) Quantification of lesion lengths at 36 and 48 hpi. Different letters indicate statistically significant differences (*p* < 0.05).


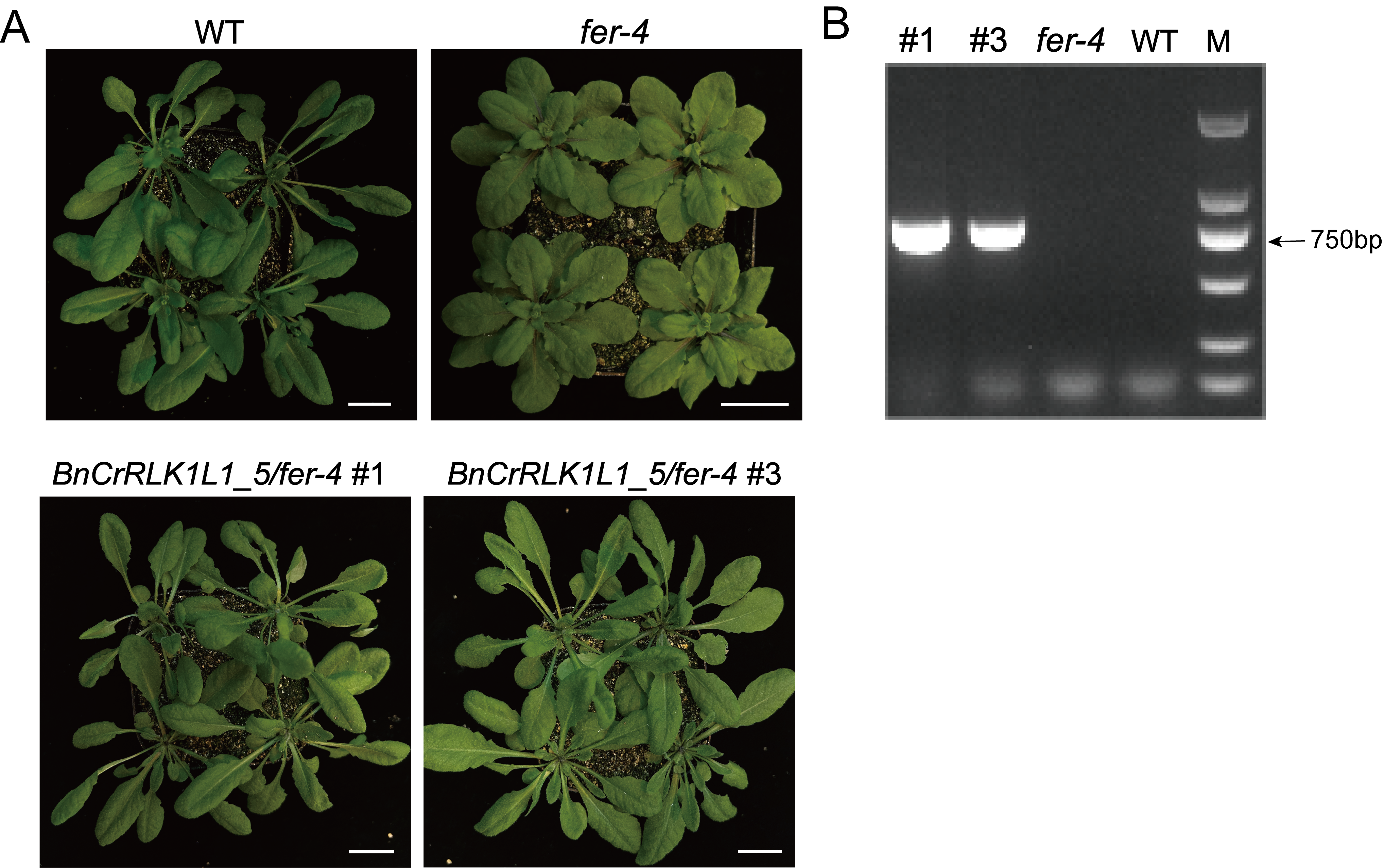


**Fig. S5 Molecular identification and phenotypic observation of *BnCrRLK1L1_5* complementation lines.** (A) Representative aboveground phenotypes of 6-week-old wild-type (WT), *fer-4* mutant, and two independent complementation lines. Scale bar = 2 cm. (B) PCR amplification of BnCrRLK1L1_5 transgene in complementation lines. The specific band size is approximately 750 bp. M, DNA marker.


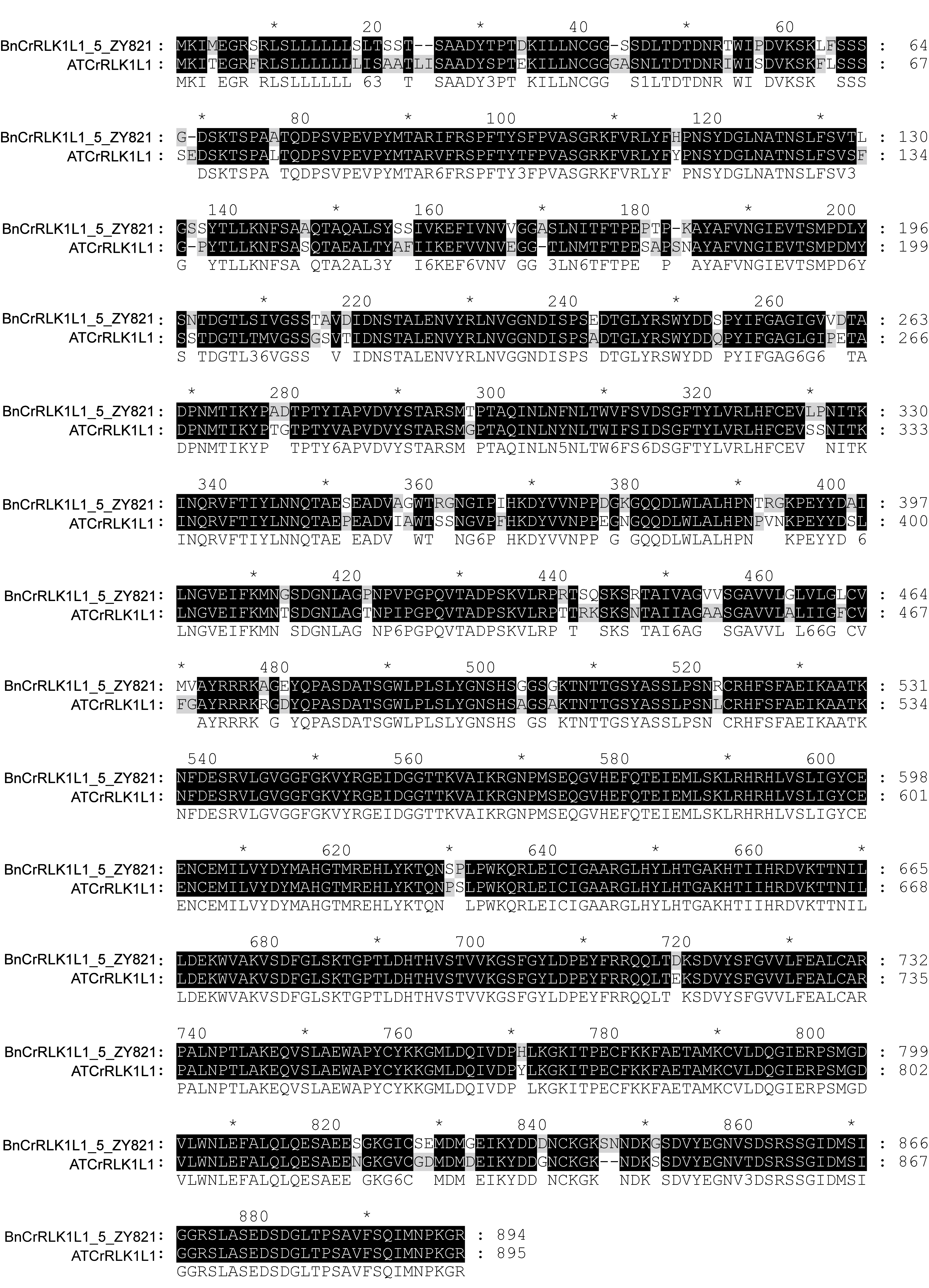


**Fig. S6 Protein sequence alignment of** **BnCrRLK1L1_5 and AtCrRLK1L1.** Identical residues are highlighted with a black background.


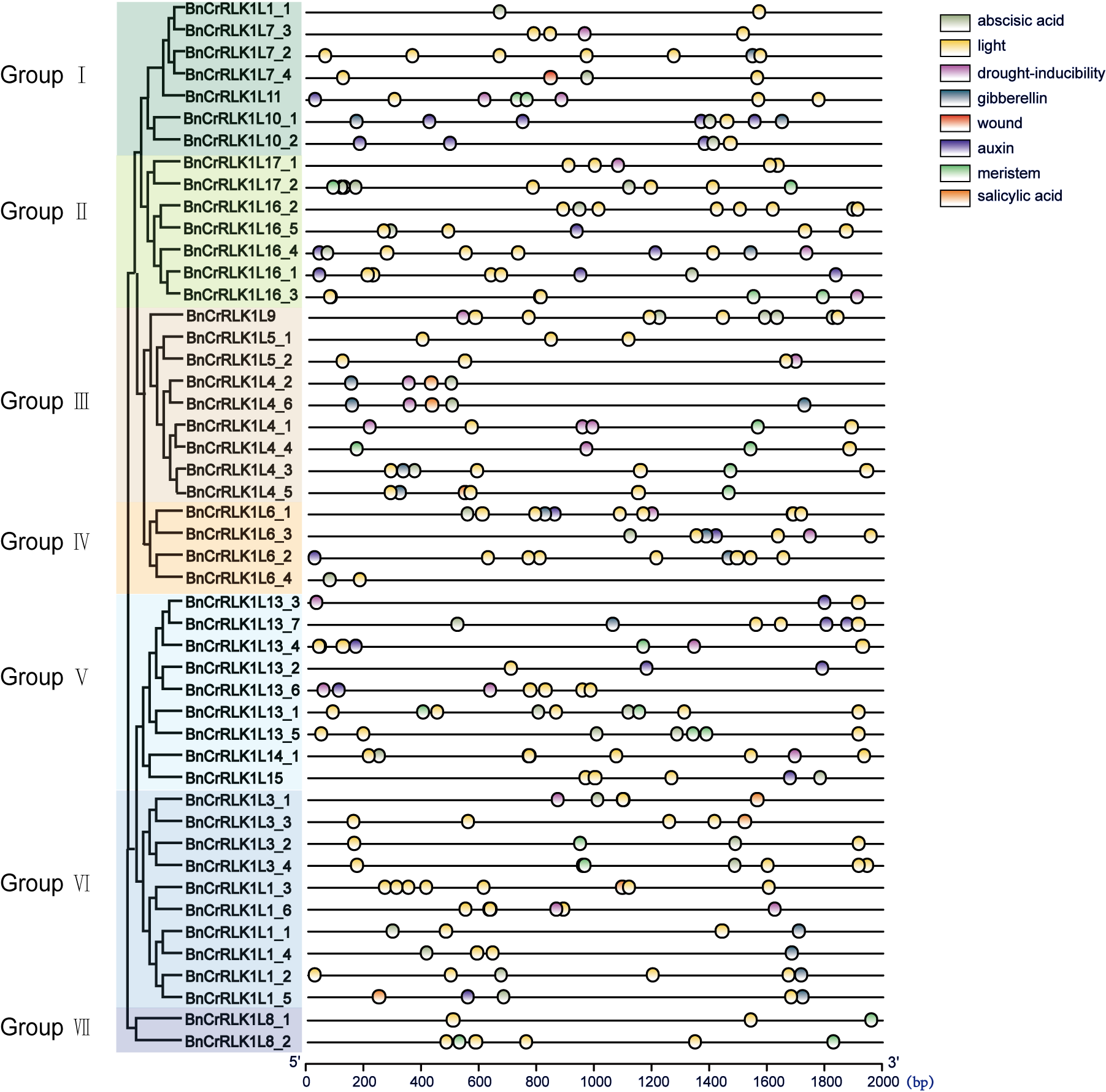


**Fig. S7 Identification, classification and localization of *cis*-elements in promoters of *BnCrRLK1L* genes*.***
